# Clinical features, risk factors and outcomes of contact lens-related bacterial keratitis in Nottingham, UK: a 7-year study

**DOI:** 10.1038/s41433-024-03323-7

**Published:** 2024-09-12

**Authors:** Lakshmi Suresh, Yasmeen Hammoudeh, Charlotte S. Ho, Zun Zheng Ong, Jessica Cairns, Bhavesh P. Gopal, Lazar Krstic, Ahmad Elsahn, Michelle M. Lister, Dalia G. Said, Harminder S. Dua, Darren S. J. Ting

**Affiliations:** 1 Academic Ophthalmology, School of Medicine, University of Nottingham, Nottingham, UK; 2 Department of Ophthalmology, Western Eye Hospital, London, UK; 3 New Cross Eye Hospital, Wolverhampton, UK; 4 Department of Ophthalmology, Luton Hospital, Luton, UK; 5 Department of Ophthalmology, Queen’s Medical Centre, Nottingham, UK; 6 Department of Ophthalmology, King’s Mill Hospital, Sherwood Forest Hospitals NHS Foundation, Sutton-in-Ashfield, UK; 7 Department of Microbiology, Queen’s Medical Centre, Nottingham, UK; 8 Academic Unit of Ophthalmology, Institute of Inflammation and Ageing, University of Birmingham, Birmingham, UK; 9 Birmingham and Midland Eye Centre, Birmingham, UK

**Keywords:** Corneal diseases, Infectious diseases, Epidemiology, Outcomes research

## Abstract

**Background/Objectives:**

To examine the clinical characteristics, risk factors and outcomes of contact lens-related bacterial keratitis (CLBK) in a large UK tertiary referral centre.

**Subjects/Methods:**

A retrospective analysis of all patients who presented to the Queen’s Medical Centre, Nottingham, UK, with suspected CLBK between October 2015 to September 2022 (a 7-year period) was performed. Relevant data on demographic factors, CL wear behaviour, causes, clinical characteristics, and outcomes were analysed.

**Results:**

We included 138 patients with CLBK; the mean age was 42.0 ± 17.8 years and 74 (53.6%) patients were male. Most CLBK were related to soft CL wear (94.5%), particularly monthly disposable (42.5%) and daily disposable (24.4%) CLs. Poor CL wear behaviour/hygiene was documented in 57.1% cases. Among the 64 (46.4%) microbiological-positive cases (n = 73 organisms), *Pseudomonas aeruginosa* (36, 49.3%) and *Staphylococcus spp*. (16, 21.9%) were most commonly identified. Six (4.3%) cases were polymicrobial. Most (97.0%) patients were successfully treated with topical antibiotics alone, with 80.6% achieving good final corrected-distance-visual-acuity (CDVA) of ≥ 0.30 logMAR. Poor visual outcome (final CDVA < 0.30 logMAR) was significantly associated with presenting CDVA < 0.6 logMAR (*p* = 0.002) and central ulcer (*p* = 0.004). Poor corneal healing (complete healing of > 30 days from initial presentation) was significantly associated with age > 50 years (*p* = 0.028), female gender (*p* = 0.020), and infiltrate size >3 mm (*p* = 0.031).

**Conclusions:**

Poor CL wear behaviour/hygiene is commonly observed in CLBK, highlighting the importance of improved counselling and awareness regarding CL use and hygiene. When presented early and managed appropriately, most patients are able to achieve good clinical outcomes with medical treatment alone.

## Introduction

Infectious keratitis (IK) represents the leading cause of corneal blindness worldwide [1, 2]. Global incidence of IK has been estimated at approximately 2.5 to 799 cases per 100,000 population/year, with a considerably higher incidence in the low- and middle-income countries (LMICs). It is a painful and potentially sight-threatening ocular disease that often requires intensive treatment and/or hospitalisation, posing significant impact on patients’ quality of life, healthcare systems, and economy [3].

IK can be caused by a wide range of pathogens, including bacteria, fungi, protozoa, and viruses. Bacterial keratitis (BK) has been consistently shown to be the most common cause of microbial keratitis (i.e., bacterial, fungal, and protozoan keratitis) in developed countries, including the UK, accounting for > 90% of all cases of microbial keratitis in some studies [4, 5]. The innate defence mechanisms at the ocular surface ensure that IK rarely occurs without any predisposing factor [2, 6, 7]. Among all, contact lens (CL) wear is recognised as one of the main risk factors for BK [7–10]. Some studies have highlighted ~10-80 times increased risk of developing IK in CL wearers compared to healthy non-CL wearers [11, 12]. This has a significant implication on global health as the number of contact lens wearers has been estimated at 140 million worldwide and is likely to increase in the coming decades [13].

CL-related IK tends to affect younger patients and working adults more commonly due to their higher social and occupational needs for CL [8, 14]. This could have a considerably negative effect on work productivity and economy as a result of lost working days. CL-related IK is most commonly caused by *Pseudomonas aeruginosa*, likely attributed to its ability to survive in contact lens solution and ocular environment and to form biofilm on CL [8, 9, 15]. Several risk factors associated with CL-related IK have been highlighted; these included CL material and design, overnight wear, showering/swimming with CL use, and poor hygiene, amongst others [16–18].

Despite the prevalent number of CL wearers and the potential impact of CL-related IK, there was no study in the past decade that had specifically evaluated the clinical characteristics and outcomes of CLBK in the UK. In view of the gap in the literature, this study aimed to provide an up-to-date analysis of CL-related BK (CLBK) in a major tertiary ophthalmic referral centre in the UK.

## Materials and methods

This was a retrospective study which captured all patients who had presented to Queen’s Medical Centre, Nottingham, UK between October 2015 and September 2022 (a 7-year period) with suspected CLBK bacterial keratitis and underwent corneal sampling. The study was approved as a clinical audit by the Clinical Governance team at the Nottingham University Hospitals NHS Trust (Ref: 19–265C) and was conducted in accordance with the tenets of Declaration of Helsinki.

### Case identification, inclusion and exclusion

Potential eligible patients were first identified through the local microbiology database [8, 19]. Both microbiological-positive and microbiological-negative presumed CLBK cases were included. Microbiological-positive cases were defined by the presence of clinical characteristics of BK (e.g., corneal epithelial defect, infiltrate, and anterior chamber activity) with microbiological confirmation of bacterial organism(s) from corneal sampling. Microbiological-negative cases were defined by the presence of clinical characteristics of BK in the absence of positive microbiological result and was treated with intensive topical antibiotic treatment alone (but no other antimicrobial treatment). Exclusion criteria included cases that were not related to CL wear, non-bacterial related or mixed infections (i.e. mixed bacterial, fungal and/or Acanthamoeba infections).

### Data collection

Relevant data regarding demographic factors, risk factors, presenting features, corrected-distance-visual-acuity (CDVA), causative organisms, treatment, and outcomes were collected from local electronic health record systems using a standardised excel proforma. Details on CL wear included the type of CL worn (e.g. daily/monthly disposable soft CL, extended wear soft CL, rigid gas permeable (RGP) CL, therapeutic bandage CL, and cosmetic CL), overnight wear, contact with water (e.g. showering or swimming with CL wear), and frequency/duration of wear. Extended wear was referred to the CL type that were designed to be worn continuously, including overnight, whereas overnight wear was referred to the behavioural use of CL overnight (irrespective of whether the CL was designed as extended wear or not). The size of epithelial defect and infiltrate was defined by the maximal linear dimension of the ulcer and was divided into 3 categories: (1) small ( < 3.0 mm); (2) moderate (3.1–6 mm); and (3) large ( > 6.0 mm) [8]. The location of ulcer was defined as: (1) peripheral: ulcer located fully within 3 mm of the limbus; (2) paracentral: any part of the ulcer involving the paracentral cornea ( > 3 mm from the limbus) but not affecting the visual axis; and (3) central: any part of the ulcer affecting the visual axis.

### Microbiological investigations

All patients who presented with suspected CLBK with any of the following clinical characteristics, including (1) ulcer of >1 mm diameter, (2) central location, (3) presence of anterior chamber activity and/or hypopyon, and/or (4) atypical clinical presentation, underwent corneal sampling as per the departmental guideline [8]. Corneal samples were sent for microscopic examination, microbiological culture and susceptibility testing, and/or 16S/18S polymerase chain reaction (PCR; only introduced since June 2021) [20]. CL and CL solution were not routinely sent for culture, and therefore the results were not included in the analysis. Cultures were inoculated on chocolate, blood, fastidious anaerobic, and Sabouraud dextrose agars to isolate bacterial and fungal organisms. For suspected *Acanthamoeba* keratitis, corneal sampling was performed for either culture (using non-nutrient agar with *Escherichia coli* overlay) or PCR [21]. However, as stated, fungal and *Acanthamoeba* positive cases were excluded from this study.

### Clinical management

All patients were started on intensive topical antibiotics with either fluoroquinolone monotherapy (levofloxacin 0.5% or moxifloxacin 0.5%) or dual therapy using fortified cefuroxime 5% plus fortified aminoglycoside (e.g., amikacin 2.5% or gentamicin 1.5%) or levofloxacin 0.5%. Topical antibiotics were given every hourly for first 48 h then slowly tapered off over a few weeks-months, depending on the treatment response and clinical progress. Adjustments to treatment were made (if necessary) depending on the microbiological results and clinical response to treatment. Hospitalisation was indicated in moderate/severe or potentially sight-threatening cases, presence of considerable corneal melt or threatened/actual corneal perforation, or patients who might not manage or comply with treatment at home [8]. Systemic antibiotic was administered if there was risk or evidence of sclerokeratitis or intraocular involvement such as endophthalmitis (which would also require intravitreal antibiotics).

### Statistical analysis

Statistical analysis was performed using SPSS software version 28.0 (IBM Corp; Armonk, NY, USA). For descriptive and analytic purposes, cases were divided into microbiological-positive and microbiological-negative cases. Chi square test or Fisher’s Exact test was used to analyse the difference between categorical variables whereas Student’s T-test or Mann-Whitney U-test was used to compare the means between two groups where appropriate. CDVA was recorded and analysed in logMAR unit. CDVA of counting fingers, hand movement, perception of light, and no perception of light were converted to 1.9, 2.3, 2.8, and 3.0 logMAR, respectively [22]. Multivariable logistic regression analysis was conducted to examine for any prognostic factors for poor visual outcome (defined as a final CDVA < 0.30 logMAR) and poor corneal healing (defined as > 30 days for complete corneal healing). Odds ratios (OR) were calculated and presented with a 95% confidence interval (CI). *P*-value of < 0.05 was considered statistically significant.

## Results

A total of 138 patients (*n* = 138 eyes) were included. Patients’ mean age was 42.0 ± 17.8 years, with 120 (87.0%) being of working age (i.e. 16–64 years old) and 74 (53.6%) being male (Table 1). No bilateral cases were identified. The mean follow-up duration was 2.9 ± 5.3 months. Of all cases, 64 (46.4%) were microbiological-positive.

**Table 1:** Summary of the baseline characteristics of patients who presented to Queens Medical Centre, Nottingham, UK, with microbiological-positive (MP) and microbiological-negative (MN) contact lens-related bacterial keratitis.

	All cases; Total *N* = 138, *N* (%)	MP group; Total *N* = 64, *N* (%)	MN group; Total *N* = 74, *N* (%)	*P*-value*
Age, years	42.0 ± 17.8	42.5 ± 18.5	41.6 ± 17.3	0.77
Gender				0.56
Female	64 (46.4)	28 (43.8)	36 (48.6)	
Male	74 (53.6)	36 (56.2)	38 (51.4)	
Affected eye				0.93
Left	63 (45.7)	29 (45.3)	33 (44.6)	
Right	75 (54.3)	35 (54.7)	41 (55.4)	
Mean duration of symptoms, day	3.3 ± 5.8	3.8 ± 5.7	2.8 ± 5.8	0.31
Risk factors**				0.20
Ocular surface diseases	32 (23.2)	16 (25.0)	16 (21.6)	
Systemic immunosuppression	10 (7.2)	5 (7.8)	5 (6.8)	
Trauma	9 (6.5)	6 (9.4)	3 (4.1)	
Prior corneal surgery	5 (3.6)	5 (7.8)	0 (0.0)	
Topical steroids	5 (3.6)	4 (6.3)	1 (1.4)	
Presenting CDVA, logMAR	0.70 ± 0.76	0.97 ± 0.85	0.47 ± 0.60	< 0.001
≥ 0.3	59 (42.8)	20 (31.3)	39 (52.7)	
< 0.3–0.6	30 (21.7)	13 (20.3)	17 (23.0)	
< 0.6–1.0	18 (13.0)	8 (12.5)	10 (13.5)	
< 1.0	31 (22.5)	23 (35.9)	8 (10.8)	
Size of epithelial defect, mm^$^				0.013
< 3.0 (small)	106 (76.8)	43 (67.2)	63 (85.1)	
3.1–6.0 (moderate)	25 (18.1)	17 (26.6)	8 (10.8)	
> 6.0 (large)	7 (5.1)	4 (6.3)	3 (4.1)	
Size of infiltrate, mm^$^				0.021
1.1–3.0 (small)	116 (84.1)	49 (76.6)	69 (93.2)	
3.1–6.0 (moderate)	17 (12.3)	11 (17.2)	6 (8.1)	
> 6.0 (large)	5 (3.6)	4 (6.3)	1 (1.4)	
Location				0.10
Central	38 (27.5)	22 (34.4)	16 (21.6)	
Paracentral	74 (53.6)	34 (53.1)	40 (54.1)	
Peripheral	26 (18.8)	8 (12.5)	18 (24.3)	
Hypopyon				< 0.001
Yes	34 (24.6)	27 (42.2)	7 (9.5)	
No	104 (75.4)	37 (57.8)	67 (90.5)	
Hospitalisation required				0.002
Yes	52 (37.7)	33 (51.6)	19 (25.7)	
No	86 (62.3)	31 (48.4)	55 (74.3)	
Duration of hospitalisation, days	5.4 ± 3.3	6.4 ± 3.6	3.7 ± 1.5	0.003

*CDVA* Corrected-distance-visual-acuity

Continuous values are presented as mean ± standard deviation.

*Statistical comparison was made between culture-positive and culture-negative cases. Significant values are underlined.

**Some patients had more than one risk factor. Ocular surface diseases (*n* = 32) refer to dry eyes (*n* = 16),

^$^Statistical comparison was between the small and the moderate/large size of ulcer due to very few cases in the large group.

### Clinical features, causative organisms and antimicrobial susceptibility results

The baseline clinical characteristics are presented in Table 1. The mean duration of symptoms prior to presentation was 3.3 ± 5.8 days. The majority of the patients presented with a CDVA of ≥0.30 logMAR (i.e., 6/12 or better Snellen vision; 59, 42.8%) and an ulcer with small epithelial defect (106, 76.8%), small infiltrate (116, 84.1%), paracentral location (74, 53.6%), and absence of hypopyon (104, 75.4%). When comparing microbiological-positive and microbiological-negative cases, microbiological-positive cases had a worse presenting CDVA, larger epithelial defect and infiltrate, and higher proportion of hypopyon (all *p* < 0.05; Table 1).

Of the 64 microbiological-positive cases, 63 cases were culture-positive (including one case of smear-positive case) and one case was culture-negative but PCR-positive (which identified *P. aeruginosa* and *Abiotrophia defective*). Of all 73 bacteria isolated, *P. aeruginosa* (36, 49.3%) and *Staphylococcus spp*. (16, 21.9%) were the most common bacteria (Table 2). There were six (4.3%) cases of polymicrobial infection (i.e. infection caused by more than one bacterial species) identified. Gram-positive bacteria demonstrated excellent susceptibility to vancomycin (100%) and aminoglycosides (94.1%–100%), and moderate-to-good susceptibility to penicillin (84.6%) and fluoroquinolones (50%–77.8%). Gram-negative bacteria exhibited excellent susceptibility to aminoglycosides (95.2%–100%) and moderate-to-good susceptibility to fluoroquinolones (75%) and cefuroxime (60.0%; Supplementary Table 1).

**Table 2:** Summary of causative organisms of contact lens-related bacterial keratitis presented to the Queen’s Medical Centre, Nottingham, UK.

Organisms^a^	Total *N* = 73, *N* (%)
Gram-positive	26 (35.6)
*Staphylococcus spp*.	16 (21.9)
*Propionibacterium spp*.	7 (9.6)
*Streptococcus spp*.	3 (4.1)
Gram-negative	47 (64.4)
*Pseudomonas aeruginosa*	36 (49.3)
*Serratia spp*.	5 (6.8)
*Moraxella spp*.	3 (4.1)
Others	3 (4.1)

^a^73 organisms were identified, exceeding the total number of 64 microbiological-positive cases due to 6 cases of polymicrobial infection (all bacteria-related).

### Details of contact lens wear and risk factors

Details of CL wear, including the types of CL and behavioural risk factors, are detailed in Table 3. Based on the available information, the most common type of CL was monthly disposable refractive CL (56, 40.6%) followed by daily disposable refractive correcting lenses (30, 21.7%). A small number of cases were related to the use of RGP CL (7, 5.5%), therapeutic/bandage CL (3, 2.4%), and cosmetic CL (1, 0.9%). None of the cases was related to orthokeratology RGP CL. There was a lack of information on the types of CL material as these data were not routinely recorded in medical case notes.

**Table 3:** Summary of contact lens (CL) type and wearing behaviour in patients who presented with microbiological-positive (MP) and microbiological-negative (MN) contact lens-related bacterial keratitis to Queen’s Medical Centre, Nottingham, UK.

Parameters	All cases, *N* (%)	MP group; *N* (%)	MN group; *N* (%)	*P*-value**
CL type*^#^	127 (100.0)	58 (100.0)	69 (100.0)	0.70
Refractive – Soft CL	116 (91.3)	52 (89.7)	64 (92.8)	
Daily disposable	31 (24.4)	13 (22.4)	18 (26.1)	
1-2-weekly disposable	11 (8.7)	4 (6.9)	7 (10.1)	
Monthly disposable	54 (42.5)	25 (43.1)	29 (42.0)	
Extended wear	9 (7.1)	3 (5.2)	6 (8.7)	
Not specified	11 (8.7)	7 (12.1)	4 (5.8)	
Refractive – RGP CL	7 (5.5)	4 (6.9)	3 (4.3)	
Therapeutic / bandage	3 (2.4)	1 (1.7)	2 (2.9)	
Cosmetic	1 (0.9)	1 (1.7)	0 (0.0)	
Duration of CL wear, hours/day*	95 (100.0)	44 (100.0)	51 (100.0)	0.54
< 8	18 (18.9)	7 (15.9)	11 (21.6)	
8–12	33 (34.7)	15 (34.1)	18 (35.3)	
12–16	18 (18.9)	7 (15.9)	11 (21.6)	
> 16	26 (27.4)	15 (34.1)	11 (21.6)	
Overnight wear*	119 (100.0)	54 (100.0)	65 (100.0)	0.85
Yes	43 (36.1)	20 (37.0)	23 (35.4)	
No	76 (63.9)	34 (63.0)	42 (64.6)	
Contact with water*	119 (100)	54 (100.0)	63 (100.0)	0.65
Yes	59 (49.6)	28 (51.9)	31 (49.2)	
No	60 (50.4)	26 (48.1)	34 (50.8)	

*RGP* Rigid gas-permeable

*Some cases were excluded from analysis due to incomplete / missing details, hence the total number is different from each parameter.

^#^Comparison was made at the first level of types of CL.

^**^Statistical comparison was made between microbiological-positive and microbiological-negative cases.

Of the 119 patients with details of CL wear, 68 (57.1%) patients were noted to have at least one behavioural risk factor, including overnight wear (43, 36.1%), contact with water (59, 49.6%) and long duration of CL wear ( > 16 hours/day; 26/95, 27.4%). In addition to CL wear, 41 (29.7%) cases had at least one additional risk factor, including ocular surface disease (32, 23.2%), systemic immunosuppression/diabetes (10, 7.2%), trauma (9, 6.5%), previous corneal graft (5, 3.6%), and concomitant use of topical steroids (5, 3.6*%)*.

### Clinical management and outcomes

Full follow-up details were available for 134 (97.1%) patients. The majority (130, 97.0%) of patients were successfully treated with intensive topical antibiotics alone, including levofloxacin / moxifloxacin monotherapy (65, 48.5%) and dual therapy (69, 51.5%), consisting of cefuroxime in combination with amikacin, gentamicin, or levofloxacin. Topical steroids were used in 55 (41.0%) cases, with a mean start time of 11.1 ± 11.6 days after the initial presentation. Topical steroids were used in both microbiological-positive (31, 50%) and microbiological-negative (24, 33.3%) cases for a mean duration of 23.6 ± 20.3 days, after excluding cases that required long-term topical steroid use (e.g. eyes with previous keratoplasty) from the analysis. Hospitalisation for intensive treatment was warranted in 52 (37.7%) patients, with microbiological-positive cases having a greater need and longer duration of hospitalisation (p < 0.01) (Table 1). Three (2.2%) patients underwent optical penetrating keratoplasty for treating post-infection corneal scar and one (0.7%) patient required amniotic membrane grafting for managing persistent corneal epithelial defect. Recurrence of infection and corneal melt were noted in 11 (8.2%) and 6 (4.5%) cases, respectively, but were successfully managed with topical antibiotics. No significant adverse event, such as corneal perforation or uncontrollable infection requiring therapeutic keratoplasty or anophthalmic surgery, was noted.

**Fig. 1:**
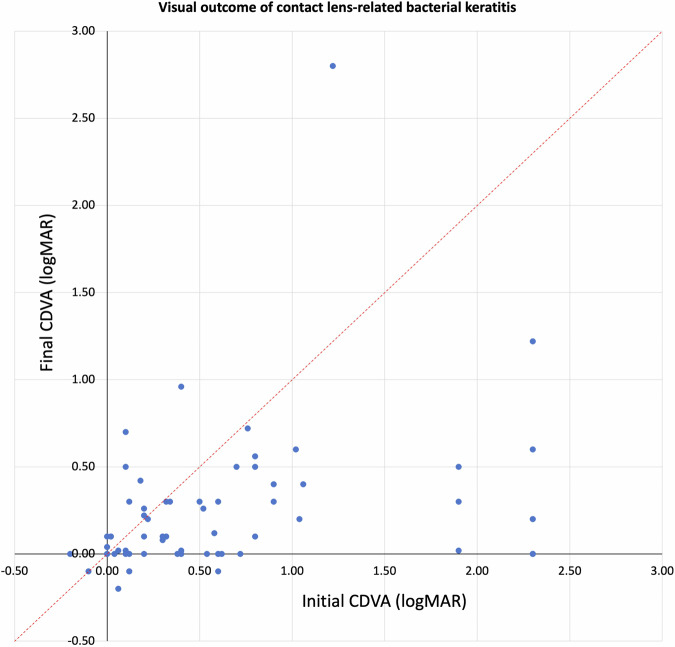
Visual outcome of contact lens-related bacterial keratitis (CLBK). A scatterplot demonstrating the corrected-distance-visual-acuity (CDVA) of CLBK at initial presentation and final follow-up visit. The dotted red line (x = y) represents no change in the visual acuity. The points located below or above the dotted red line represent an improvement or decrease in the final CDVA, respectively, when compared to the initial CDVA.

Of the 134 patients, the mean CDVA improved significantly from 0.70 ± 0.76 logMAR at presentation to 0.21 ± 0.40 logMAR at final follow-up (*p* < 0.001), with 114 (85.1%) patients achieving an equal or better final CDVA compared to the initial CDVA (Fig. 1). The proportion of patients with CDVA of ≥ 0.30 logMAR increased from 42.5% to 80.6% (*p* < 0.001). The mean complete corneal healing time was 21.4 ± 15.2 days, with 31 (23.1%) cases having a delayed corneal healing (i.e. > 30 days).

### Prognostic factors

Multivariable logistic regression analysis demonstrated that poor visual outcome was significantly influenced by presenting CDVA < 0.6 logMAR [OR 5.63 (95% CI, 1.85–17.17); *p* = 0.002] and centrally located ulcer [OR 4.53 (95% CI, 1.62–12.71); *p* = 0.004] (Table 4). Poor corneal healing time was significantly associated with age >50 years [OR 2.85 (95% CI, 1.12–7.25); *p* = 0.028], female gender [OR 3.08 (95% CI, 1.19–7.94); *p* = 0.020], and infiltrate >3 mm diameter [OR 3.53 (95% CI, 1.12–11.09); *p* = 0.031]. Other factors such as eye laterality, presence of hypopyon and positive microbiological results did not significantly affect the visual outcome or corneal healing time (all *p* > 0.05).

**Table 4:** Prognostic factors for poor visual outcome [defined as corrected-distance-visual-acuity (CDVA) of < 0.30 logMAR] and poor corneal healing (defined as >30 days to achieve complete healing or occurrence of corneal perforation or uncontrolled infection) in contact lens-related bacterial keratitis.

	Poor visual outcome		Poor corneal healing	
Parameters	Odd ratio (95% CI)	*P*-value*	Odd ratio (95% CI)	*P*-value*
Age > 50 years	0.64 (0.22–1.85)	0.40	2.85 (1.12–7.25)	0.028
Female gender	1.09 (0.41–2.92)	0.86	3.08 (1.19–7.94)	0.020
Right eye	1.23 (0.45–3.35)	0.69	2.03 (0.78–5.29)	0.15
Presenting CDVA < 0.6	5.63 (1.85–17.17)	0.002	1.27 (0.47–3.46)	0.64
Infiltrate size > 3 mm	2.13 (0.52–8.85)	0.30	3.53 (1.12–11.09)	0.031
Central ulcer	4.53 (1.62–12.71)	0.004	1.50 (0.55–4.07)	0.43
Presence of hypopyon	1.05 (0.27–4.01)	0.95	0.85 (0.27–2.63)	0.77
Positive microbiological results	0.33 (0.11–1.02)	0.06	1.71 (0.65–4.50)	0.28

^*^Multivariable logistic regression analysis was performed. Significant *p*-values are underlined.

## Discussion

CL wear represents one of the most common risk factors for IK in developed countries, accounting for 30–65% of all IK cases [1, 7, 8]. The incidence of CL-related IK was estimated at 10–130 per 100,000 people-year, highlighting its potential burden on global health and economy [1, 11, 23]. To the best of our knowledge, this study represents the largest UK study that had specifically examined the clinical characteristics, risk factors, causes, and outcomes of CLBK over the past decade.

### Demographic and risk factors

Majority (87%) of our patients were of working adult age, which was similarly observed in other studies (usually ~25–55 years old) [1, 8, 16, 24, 25]. Some studies have identified an increase in incidence of CL-related IK in younger patients ( ~ 20–30 years old), potentially related to poorer CL hygiene and use of cosmetic CLs [17, 26]. A range of modifiable risk factors have previously reported, including extended wear, overnight wear, contact with water, poor CL storage case hygiene/care, and poor hand hygiene. [16–18, 23, 24] In our study, we noted poor CL wear behaviours/hygiene such as overnight wear, extended wear and contact with water in > 50% of our patients, and this figure may be potentially underestimated due to possible under-recording in clinical notes. Furthermore, the type of CL serves as another significant influencing factor for CL-related IK [12, 18, 23]. Extended wear soft CL has been shown to have the highest risk of CL-related IK, followed by daily soft CL (non-extended wear) and rigid gas-permeable CL [15, 23, 24, 27, 28]. Our study showed that monthly disposable CL was the most common type (42.5%) of CL whereas extended wear CL was the least common choice (7.1%). In view of the low number of CLBK cases associated with extended overnight wear CL (despite its inherent higher risk of causing infection), one may infer a reducing trend in the use of extended overnight wear CL in this UK population. While all these risk factors increase the risk of CLBK, we did not observe any significant influence of these risk factors on microbiological positivity.

### Clinical features and causative organisms

Majority of our cases were of mild severity (84% with < 3 mm infiltrate), which might be attributed to the early presentation to the hospital (the mean duration of symptoms before presentation was 3.3 days). This has significant clinical implications as visual outcome of IK, including CLBK, is highly dependent on timely diagnosis and treatment [8, 29]. The relatively mild severity of infection may also be attributed to the low proportion ( < 10%) of extended wear CL in our cohort. Extended wear CL can increase the retention time and growth of the organisms on the ocular surface, resulting in more severe infection, as opposed to daily replacement of CL [24]. We also observed that microbiological positivity was correlated with the severity of infection, which was similarly observed in previous studies [8, 30]. In contrast, a US study conducted more than a decade ago reported that a considerably higher proportion of CLBK patients presented with more severe infection, with an ulcer > 4 mm in size (46%) and hypopyon (36%) [31]. The disparity in severity may be related to the difference in the proportion of cases affected by *P. aeruginosa* (63% in the US study), ocular co-morbidities, and/or time interval between onset of symptoms and presentation to the hospital.

*P. aeruginosa* (49.3%) and *Staphylococcus spp*. (21.9%) were shown to be the most common organisms in our study, paralleling the findings of many other studies [9, 16, 24, 28, 31]. However, a study conducted in South India identified *Serratia spp*. as the most common organism for CLBK, whereas a Japanese study reported *Staphylococcus epidermidis* as the most common causative organism [9, 25]. Such heterogeneity highlights the geographical and temporal variations of the disease and the importance of up-to-date regional analysis of the causative organisms (as it may serve as a useful guide for treatment, particularly when the microbiological result is negative).

### Clinical and visual outcomes

The majority (97.0%) of our patients were successfully managed with topical antibiotics alone (without systemic antibiotic or surgical intervention), with 80.6% achieving a final CDVA of ≥ 0.30 logMAR. Enzor et al. [32] reported a slightly higher rate of surgical intervention (14%) for their patients with CLBK. However, they only included cases affected by *P. aeruginosa*, which is notoriously known to cause severe corneal infection, stromalysis and visual loss [9, 28, 32]. Nonetheless, CLBK has been shown to have a better visual outcome than non-CLBK [32]. This may be attributed to a younger age, less ocular/systemic comorbidities and earlier presentation of the disease in patients with CLBK (as CL wearers may be more aware/concern of any new ocular symptoms that can interfere with their CL wear). Indeed, when compared to our previous BK study (which included 65% non-CLBK and 35% CLBK) [8], the mean duration of symptoms prior to presentation was 5.3 days in the previous study (as opposed to 3.3 days in this study), suggesting that CL wearers are more likely to seek earlier medical attention. In addition, 35% of the patients in the previous study had more severe infection at presentation than this study (35% vs. 16% with ulcers of >3 mm infiltrate).

We also observed that poor visual outcome was significantly associated with poorer presenting CDVA and central ulcer. These findings were similarly observed in other CLBK studies conducted in France and the US [10, 32], highlighting the importance of prompt diagnosis and treatment in CLBK and IK in general [16]. Furthermore, we found that corneal healing was negatively correlated with increased age and larger infiltrate size. Increased cell senescence in ageing cornea may affect the epithelial adhesion molecules function, phagocytic ability of reactive polymorphonuclear cells, and the innate and/or adaptive immune responses at the ocular surface, resulting in slower eradication of infection and delayed corneal healing [33]. In addition, older patients generally have multiple ocular/systemic co-morbidities (e.g. ocular surface diseases, diabetes, previous corneal surgeries, etc.), which can contribute to poor wound healing. Interestingly, female gender was associated with poorer corneal healing, which contrasted the findings observed by Das et al. [25] and Konda et al. [27]. While sex-specific difference in corneal healing has been shown in mice studies (with female mice corneas having a slower healing rate due to the effect of oestradiol) [34], this observation has not been supported by other preclinical or human corneal studies [35]. Our finding may be confounded by other variables (e.g., difference in types and severity of co-morbidities) and requires further elucidation.

While we found that microbiological positivity was associated with more severe infection, it did not influence the visual outcome or the corneal healing time, similarly observed in other studies [31, 36]. One plausible explanation is the ability to initiate/administer the optimal antibiotic therapy based on the positive microbiological and susceptibility/resistance results, thereby achieving a similarly good outcome as microbiological-negative cases. In addition, a significantly higher proportion of patients with microbiological-positive CLBK were hospitalised for intensive treatment and monitoring (for a longer duration), which might have contributed to the good clinical recovery and outcomes. Reassuringly, only a few patients required surgical interventions, including amniotic membrane grafting, which has been shown to expedite corneal healing in bacterial keratitis [37].

### Study limitations

One of our study limitations is that we only included cases that had undergone corneal sampling; hence some of the very mild CLBK cases (i.e. small ( < 1 mm), non-sight-threatening ulcer) might not have been captured by this study. However, these very mild cases usually have less impact on the patients and are responsive to topical antibiotics; if not, the affected patients would re-present to our unit for further management (which would include corneal sampling and be included in our study). Another limitation is the inclusion of microbiological-negative CLBK cases. However, all cases were only included after careful analysis of patients’ case notes to confirm eligibility. In addition, microbiological-negative cases represent >50% of all IK cases in the real-world setting in many regions, including the UK [5, 19]. Therefore, a good understanding of the clinical characteristics, management and outcomes of microbiological-negative cases are invaluable for clinicians and patients. Implementation of molecular diagnostics and next-generation sequencing may potentially ameliorate the low culture yield in the future [38–40]. As this study was not a case-control study, we could not ascertain the relative/absolute risk difference of CLBK among each CL type. It would also be interesting to analyse the impact of CL material on the clinical characteristics of CLBK as it has been shown to influence the severity of infection [18], though these data were not routinely collected in daily clinical practice. Nonetheless, we observed that 57% of our patients reported poor CL hygiene, and this figure might be underestimated as these risk factors might be under-recorded in medical notes.

In conclusion, CLBK represents as a significant ocular condition that can have a negative impact on patient’s vision, quality of life, healthcare resources, and work productivity (as affected patients are usually of working age). Timely presentation, diagnosis and treatment is key to achieving a good outcome. Poor CL behavioural risk factors are commonly observed among our patients, highlighting the need for improved education and awareness of CL care/hygiene.

## Summary

### What was known before

- Contact lens (CL) wear is a major risk factor for bacterial keratitis.
- Poor CL wear behaviour/hygiene increases the risk of CL-related bacterial keratitis (CLBK).
- There is no study in the UK that had specifically analysed the risk factors, causes and outcomes of CLBK in the past decade.

### What this study adds

- This study provides one of the most comprehensive and up-to-date analyses of CLBK in the UK in the past decade.
- With prompt diagnosis and treatment, most patients with CLBK can be successfully treated with intensive topical antibiotics alone and achieve a good outcome.
- Microbiological positivity is significantly associated with the initial severity of CLBK but has no significant influence on the final visual outcome or corneal healing time.

## Supplementary information

Supplementary Table 1.

## Data Availability

The authors confirm that the data supporting the findings of this study are available within the article and its supplementary materials.
