# Supplementary material for: Clinical features, risk factors and outcomes of contact lens-related bacterial keratitis in Nottingham, UK: a 7-year study
Source: Eye (Lond). 2024 Sep 12;38(18):3459–66. doi: 10.1038/s41433-024-03323-7 (PMC11621543; doi:10.1038/s41433-024-03323-7)
Supplement: Supplementary file 1 — Supplementary Table 1. [file 41433_2024_3323_MOESM1_ESM.docx]

**Supplementary Table 1.** Summary of antibiotic susceptibility of 71 causative organisms of culture-positive contact lens-related bacterial keratitis presented to the Queen’s Medical Centre, Nottingham, UK.

| **Organisms** | **Antibiotics*** | | | | | | |
| --- | --- | --- | --- | --- | --- | --- | --- |
|  | **Penicillin^#^** | **Cefuroxime** | **Amikacin** | **Gentamicin** | **Ciprofloxacin** | **Levofloxacin** | **Vancomycin** |
| **Gram-positive** | 22/26 (84.6) | - | 2/2 (100.0) | 16/17 (94.1) | 8/16 (50.0) | 7/9 (77.8) | 15/15 (100.0) |
| *Staphylococcus spp.* | 12/16 (75.0) | - | 2/2 (100.0) | 15/16 (93.8) | 8/16 (50.0) | 6/8 (75.0) | 9/9 (100.0) |
| *Propionibacterium spp.* | 7/7 (100.0) | - | - | - | - | 1/1 (100.0) | 6/6 (100.0) |
| *Streptococcus spp.* | 3/3 (100.0) | - | - | 1/1 (100.0) | - | - | - |
| **Gram-negative** | 3/9 (33.3) | 3/5 (60.0) | 42/42 (100.0) | 40/42 (95.2) | 33/44 (75.0) | 33/44 (75.0) | - |
| *Pseudomonas aeruginosa* | - | - | 35/35 (100.0) | 34/35 (97.1) | 25/35 (71.4) | 25/35 (71.4) | - |
| *Serratia spp.* | 0/5 (0) | 0/1 (0) | 5/5 (100.0) | 4/5 (80.0) | 4/5 (80.0) | 4/5 (80.0) | - |
| *Moraxella spp.* | 1/2 (50.0) | 2/2 (100.0) | - | - | 2/2 (100.0) | 2/2 (100.0) | - |
| *Others ** | 2/2 (100.0) | 1/2 (50.0) | 2/2 (100.0) | 2/2 (100.0) | 2/2 (100.0) | 2/2 (100.0) | - |

*Based on the local microbiology laboratory protocol, some antibiotics were only tested against a particular type of organism.

^#^Penicillin group includes penicillin, amoxicillin, and flucloxacillin.

*Includes *E. Coli and Acinetobacter spp.*
